# Effects of integrated intrinsic foot muscle exercise with foot core training device on balance and body composition among community-dwelling adults aged 60 and above

**DOI:** 10.1186/s12877-024-04945-y

**Published:** 2024-05-07

**Authors:** Sheng-Lun Kao, Mei-Lan Hsiao, Jen-Hung Wang, Chen-Sheng Chen, Shin-Yuan Chen, Yung-Jeng Shiau, Chich-Haung Yang

**Affiliations:** 1 Department of Family Medicine, Hualien Tzu Chi Hospital, Buddhist Tzu Chi Medical Foundation, No. 707, Sec. 3, Chung Yang Rd., Hualien, 970 Taiwan; 2 Department of Family Medicine, College of Medicine, Tzu Chi University, No. 701, Sec. 3, Chung Yang Rd., Hualien, 970 Taiwan; 3 Department of Nursing, Hualien Tzu Chi Hospital, Buddhist Tzu Chi Medical Foundation, No. 707, Sec. 3, Chung Yang Rd., Hualien, 970 Taiwan; 4 Department of Medical Research, Hualien Tzu Chi Hospital, Buddhist Tzu Chi Medical Foundation, No. 707, Sec. 3, Chung Yang Rd., Hualien, 970 Taiwan; 5 Department of Physical Therapy and Assistive Technology, National Yang Ming Chiao Tung University, No. 155, Sec. 2, Linong St., Beitou District, Taipei, 112 Taiwan; 6 Department of Neurosurgery, Hualien Tzu Chi Hospital, Buddhist Tzu Chi Medical Foundation, No. 707, Sec. 3, Chung Yang Rd., Hualien, 970 Taiwan; 7 School of Medicine, Tzu Chi University, No. 701, Sec. 3, Chung Yang Rd., Hualien, 970 Taiwan; 8 Department of Physical Therapy, College of Medicine, Tzu Chi University, No. 701, Sec. 3, Chung Yang Rd., Hualien, 970 Taiwan; 9 Sports Medicine Center, Hualien Tzu Chi Hospital, Buddhist Tzu Chi Medical Foundation, No. 707, Sec. 3, Chung Yang Rd., Hualien, 970 Taiwan

**Keywords:** Older adult, Intrinsic foot muscle, Balance, Mobility, Body composition

## Abstract

**Background:**

Evidence on the effects of plantar intrinsic foot muscle exercise in older adults remains limited. This study aimed to evaluate the effect of an integrated intrinsic foot muscle exercise program with a novel three-dimensional printing foot core training device on balance and body composition in community-dwelling adults aged 60 and above.

**Methods:**

A total of 40 participants aged ≥ 60 years were enrolled in this quasi-experimental, single-group, pretest-posttest design; participants were categorized into two groups, those with balance impairment and those without balance impairment. The participants performed a 4-week integrated intrinsic foot muscle exercise program with a three-dimensional printing foot core training device. The short physical performance battery (SPPB) and timed up and go test were employed to evaluate mobility and balance. A foot pressure distribution analysis was conducted to assess static postural control. The appendicular skeletal muscle mass index and fat mass were measured by a segmental body composition monitor with bioelectrical impedance analysis. The Wilcoxon signed rank test was used to determine the difference before and after the exercise program.

**Results:**

Among the 40 enrolled participants (median age, 78.0 years; female, 80.0%; balance-impaired group, 27.5%), the 95% confidence ellipse area of the center of pressure under the eyes-closed condition was significantly decreased (median pretest: 217.3, interquartile range: 238.4; median posttest: 131.7, interquartile range: 199.5; *P* = 0.001) after the exercise. Female participants without balance impairment demonstrated a significant increase in appendicular skeletal muscle mass index and a decrease in fat mass. Participants in the balance-impaired group exhibited a significant increase in SPPB.

**Conclusions:**

Integrated intrinsic foot muscle exercise with a three-dimensional printing foot core training device may improve balance and body composition in adults aged 60 and above.

**Trial registration:**

ClinicalTrials.gov ID: NCT05750888 (retrospectively registered 02/03/2023).

## Background

The aging process is universally recognized as a phenomenon that introduces physiological changes affecting various aspects of health and well-being [1]. Among the elderly, maintaining a specific balance and body composition becomes crucial. This is especially pertinent as these factors directly impact overall health, mobility, and quality of life in older adults [2]. The intrinsic foot muscles (IFMs) play a significant role in this context, being key foot stabilizers involved in passive, active, and neural subsystems that constitute the foot core system [3, 4]. IFMs contribute to arch support, shock absorption, and provide afferent information essential for the gross movement executed by extrinsic foot muscles [3, 5]. However, aging leads to weaker toe strength and foot muscles in older adults compared to their younger counterparts [6, 7]. Such weaknesses have been linked to imbalances and decreased functional mobility in the elderly [8].

Isolated intrinsic foot strengthening exercises, including toe yoga, toe-flexion exercises, and short foot exercises (SFEs), have demonstrated efficacy in improving muscle strength, balance, and functional mobility among older adults [9]. SFEs, involving the isolated contraction of IFMs, have proven more effective than other exercises in activating IFMs and enhancing dynamic balance [10, 11]. To perform SFEs effectively, individuals attempt to elevate the arch and subsequently shorten the length of the foot by drawing the heads of the metatarsals toward the calcaneus while avoiding extrinsic motion [12]. However, a significant challenge lies in providing effective instruction and learning methods for these exercises, especially in the context of geriatric rehabilitation. Recognizing this challenge, the study at hand addresses the need for improved training methods by introducing a novel three-dimensional (3-D) printed foot core training device. This device is designed to enhance the effectiveness of IFM training, offering features such as customization, ease of modification, and improved efficacy.

The primary aim of this study is to investigate the feasibility of an integrated IFM strengthening exercise program for community-dwelling adults aged 60 and above. This involves the utilization of a unique intrinsic foot muscle strengthening device developed using 3-D printing techniques. The study assesses the short-term (4-week) effects of this integrated IFM exercise program on balance and body composition in community-dwelling adults aged 60 and above.

## Methods

### Study design and setting

This study adopted a quasi-experimental single-group pretest-posttest design and was conducted in the community care center of Hualien County, Taiwan. We employed a 4-week integrated IFM exercise program with 3-D printing foot core training devices. Measures of physical performance, including short physical performance battery (SPPB), timed up and go (TUG) test, foot pressure distribution, and body composition (e.g., fat mass, appendicular skeletal muscle mass [ASM]), were obtained before and after the exercise program.

### Study population

The population of the community care center included 67 individuals aged ≥ 60 years. The participants were eligible if they were 60 to 90 years old and able to engage in recreational activities. The participants were excluded if they had any history of a recent ankle or foot sprain, lower extremity fracture, or disability due to systemic diseases that severely affected balance and mobility. The participants who met the inclusion criteria provided signed informed consent. The study was approved by the Institutional Review Board (IRB) of Hualien Tzu Chi Hospital (IRB no. 110-084-A).

### Exercise program and data collection

The participants provided demographic information and completed the Barthel Index for activities of daily living (ADLs) [13] and Mini-Mental State Examination [14]. The body weight, fat mass, and ASM were measured by a segmental body composition monitor with bioelectrical impedance analysis (BC-545 N, Tanita, Tokyo, Japan). The body mass index was calculated as weight in kilograms divided by height in meters squared. The ASM index (ASMI) was defined as ASM (kg)/height^2^ (m^2^) [15].

We employed the SPPB and TUG tests to evaluate mobility and balance. The SPPB is an objective test that uses three tests to measure physical performance: static balance in three positions (side-by-side, semi-tandem, and tandem stand), 4-meter gait speed, and five times chair stand test. The total SPPB score ranges from 0 (worst) to 12 (best) [16]. The TUG test is a simple test that measures functional mobility and fall risk. In this test, individuals are asked to stand up from a chair, walk 3 m, turn 180 degrees, walk back, and sit on the chair; a faster time indicates better mobility [17].

The foot pressure distribution analysis requires participants to perform a quiet standing task on a force measuring plate (Zebris FDM-S, Zebris Medical GmbH, Germany) to measure the trajectory of the center of pressure (COP) under the eyes-closed condition; results are used to assess static postural control. We selected COP variables, including 95% confidence ellipse area (sway area), total sway length of COP, and average velocity of COP [18, 19]. A 95% confidence ellipse area is a commonly used positional variable for calculating the scatter of COP [20]. An increase in the confidence ellipse area was associated with a significantly increased risk of falls in older adults [21]. The total sway length and average velocity of COP (defined as the total sway length divided by the duration of measurement) are two of the most common dynamic variables used to evaluate posture control after the exercise program.

**Fig. 1:**
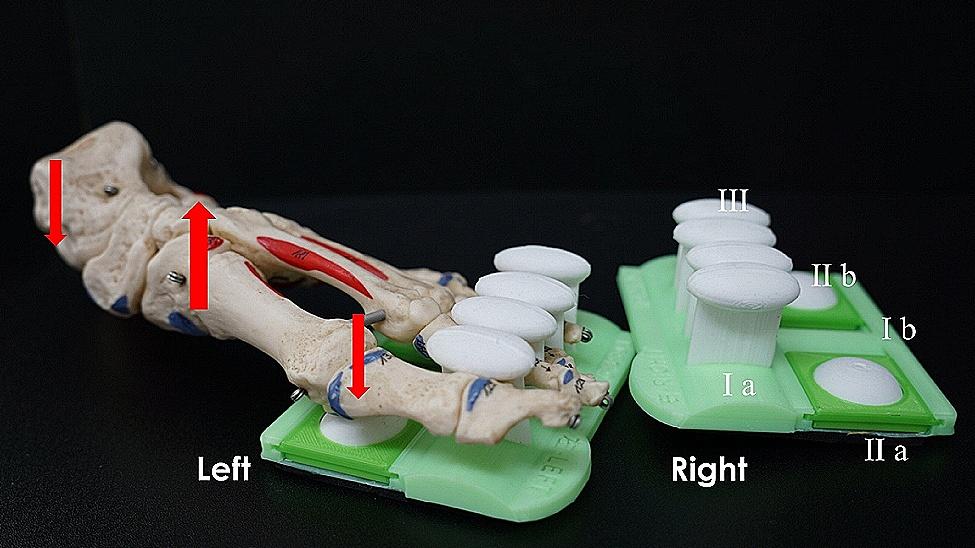
3-D printing foot core training device. This foot core training device comprises a base plate having a first (I a) and a second part (I b). The first part corresponds to the position of each toe of the foot, while the second part corresponds to the position of the foot’s arch and consists of the big toe resistance pad (II a) and the toe resistance pad (II b). At least four spacers (III) are provided on the base plate’s first part (I a) to separate the different toes of the foot. With downward force applied through the heel and metatarsals, the medial longitudinal arch naturally arches upward, causing the contraction of the foot core muscles. The spacers on the base plate prevent the toes from curling and causing external muscle force, while heel downforce avoids triggering calf contraction, thereby training the foot core muscles

All participants received verbal instructions, demonstrations, and guidance through a single practice trial of the integrated IFM exercise with a 3-D printing foot core training device by a board-certified orthopedic physical therapist. The integrated IFM exercise program included hallux extension, toe crunch, toe abduction, bilateral standing calf raise, resistance band ankle eversion, and resistance band ankle dorsiflexion (Table 1) [9]. A 3-D printing foot core training device (invented patent number: I693963 Taiwan), performed by exerting downward force from the heel and metatarsals of the foot to draw up the medial longitudinal arch and induce contraction of the foot core muscles, was also included in the exercise program (Fig. 1). Video of the IFM strengthening exercise and training device (Chinese version) can be accessed at https://www.youtube.com/watch?v=b5kGRjVLbdY. Our study spanned a four-week duration, allowing participants the flexibility to engage in the exercise routine up to five days a week, with one session per day. Caregivers at the community care center supervised exercise activities after initial instruction and completed daily training logs.

**Table 1:** Integrated intrinsic foot muscle exercise program with foot core training device

Exercise	Week 1–4
** A. Warm-up exercises**	
Massage for foot plantar area	20 s on each foot
Active ankle dorsiflexion and plantarflexion	1 × 10 reps on each foot
Active ankle inversion and eversion	1 × 10 reps on each foot
Passive forefoot range of motion exercises	1 × 10 reps on each foot
**B. Integrated intrinsic foot muscle exercise program**	
Active forefoot range of motion exercises (hallux extension, toe crunch, toe abduction)	1 × 10 reps, holding position for 5 s
Intrinsic foot muscle strengthening exercise with 3-D printing foot core training device	1 × 15 reps, holding position for 5 s
Bilateral standing calf raise	1 × 10 reps, holding position for 10 s
Resistance band ankle eversion	2 × 10 reps on each foot
Resistance band ankle dorsiflexion	2 × 10 reps on each foot
**C. Cool down exercises**	
Plantar sole rolling	30 s on each foot
Standing lunge stretch	1 × 3 reps, 30 s on each leg
Forefoot stretch	1 × 10 reps on each foot

The final data collection was performed at 4 weeks after the exercise program. Adherence was calculated as the ratio of actual exercise sessions reported on daily training logs to the total potential sessions during the 4-week period.

### Statistical analysis

We utilized a statistical power analysis program, G*Power 3, to calculate the sample size needed for detecting the difference between pretest and posttest, considering a two-sided significance level of 0.05, an effect size of 0.5, and a power of 0.8. This analysis indicated that a minimum of 34 participants was required [22].

The participants were categorized into two groups: those with balance impairment (defined as participants unable to perform a tandem stand for at least 10 s) and those without balance impairment, according to their pre-program balance measurements. We confirmed non-normal distribution of our data using the Kolmogorov-Smirnov test. We used descriptive statistics to estimate baseline demographic characteristics; the Mann–Whitney U test and Fisher’s exact test were employed to assess group differences between those with and without balance impairment. The Wilcoxon signed rank test was used to determine the difference in SPPB, TUG test, ASMI, fat mass, and foot pressure distribution before and after the exercise program. A two-tailed probability value of < 0.05 was considered statistically significant. All statistical analyses were performed using IBM SPSS Statistics for Windows version 22 (IBM Corp., Armonk, NY, USA).

## Results

Of the 67 individuals in the community care center, 15 were excluded because of disabilities caused by systemic diseases severely affecting balance and mobility. Overall, 52 individuals were eligible to participate in the exercise program; data was collected for 40 participants who completed the exercise program. The median age of participants was 78.0 years (interquartile range [IQR]: 10.5), and 32 (80.0%) were females. Eleven (27.5%) participants had balance impairment at the initial assessment. The group median adherence for the exercise program was 88.9% (IQR: 41.6). A comparison of baseline demographic characteristics, physical performance, sway area, and body composition of the participants in the groups with and without balance impairment is presented in Table 2. Participants with balance impairment were older and had worse cognitive function. Baseline ADLs and sway area were not associated with balance status. All participants were able to independently perform basic ADLs. Participants in the balance-impaired group exhibited a significantly lower SPPB score and a longer completion time for the TUG test compared to those without balance impairment.

**Table 2:** Baseline demographic characteristics of the participants

	Total	Without balance impairment	With balance impairment	
	(*n* = 40)	(*n* = 29)	(*n* = 11)	*P*-value
Age, years	78.0 (71.3, 81.8)	76.0 (69.0, 80.5)	80.0 (75.0, 88.0)	0.049*
Sex, female	32 (80.0)	22 (75.9)	10 (90.9)	0.41
ADL	100 (100, 100)	100 (100, 100)	100 (100, 100)	1.00
MMSE	26.0 (22.0, 28.0)	26.0 (24.5, 29.0)	23.0 (17.0, 26.0)	0.03*
SPPB	12.0 (10.3, 12.0)	12.0 (12.0, 12.0)	10.0 (7.0, 10.0)	<0.001*
TUG test, sec	9.0 (7.9, 11.3)	8.5 (7.7, 10.6)	11.4 (11.2, 14.3)	<0.001*
95% confidence ellipse area, mm^2^ (sway area)	217.3 (144.9, 383.3) (*n* = 39)	219.0 (136.7, 372.5) (*n* = 28)	207.5 (166.5, 406.0) (*n* = 11)	0.73
Total sway length of COP, mm	330.0 (251.3, 486.5) (*n* = 39)	335.5 (241.8, 482.2) (*n* = 28)	284.6 (270.8, 578.8) (*n* = 11)	0.98
**BMI, kg/m** ^**2**^				
Female	25.7 (22.7, 29.5)	25.2 (22.4, 29.3)	26.0 (24.0, 30.3)	0.93
Male	26.3 (25.0, 30.0)	26.5 (25.3, 30.9)	24.9 (24.9, 24.9)	0.83
**ASMI, kg/m** ^**2**^				
Female	6.8 (6.3, 7.1)	6.6 (6.2, 7.2)	7.0 (6.7, 7.2)	0.13
Male	9.6 (8.7, 10.3)	10.1 (8.6, 10.4)	9.0 (9.0, 9.0)	0.51
**Fat mass, %**				
Female	37.8 (32.4, 42.8)	37.8 (30.6, 42.5)	38.3 (33.2, 45.4)	0.50
Male	25.9 (22.3, 27.9)	25.3 (22.2, 28.1)	26.4 (26.4, 26.4)	0.83

*Note* Data are presented as median (Q1, Q3) or as number (percentage)

*P < 0.05

*ADL*, activities of daily living; *MMSE*, Mini-Mental State Examination; *SPPB*, short physical performance battery; *TUG*, timed up and go; *BMI*, body mass index; *ASMI*, appendicular skeletal muscle mass index

**Fig. 2:**
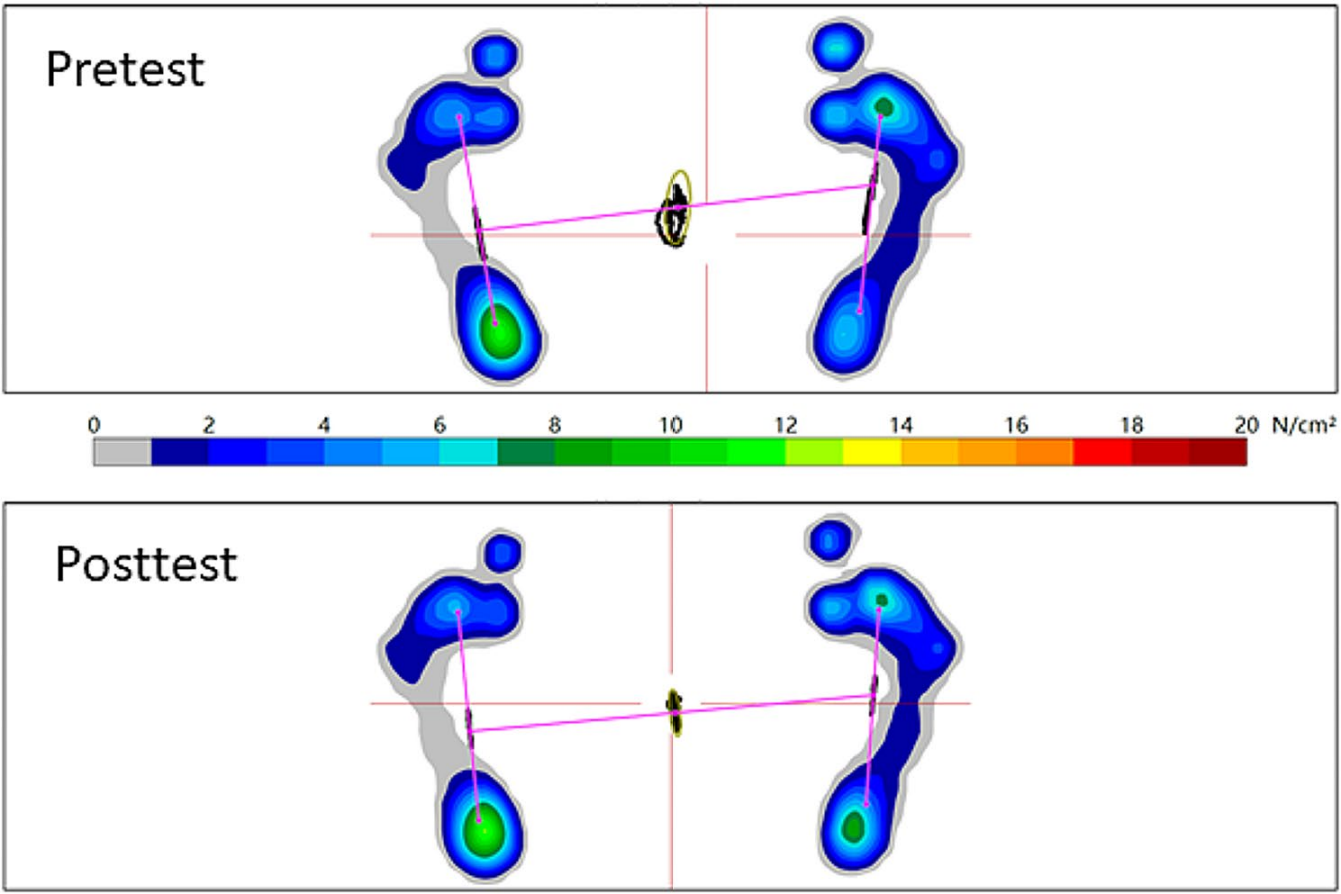
The change in foot pressure distribution and 95% confidence ellipse area of the center of pressure after the integrated intrinsic foot muscle exercise with a novel 3-D printing foot core training device

**Fig. 3:**
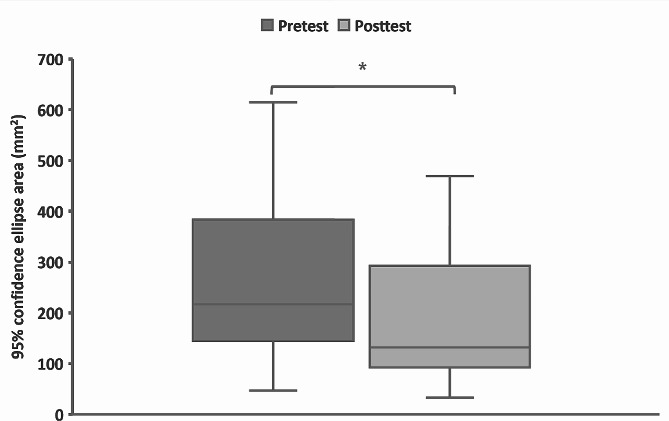
The pre-to-post measures of the 95% confidence ellipse area of the center of pressure following the integrated intrinsic foot muscle exercise with a novel 3-D printing foot core training device. *P < 0.05

Table 3 shows the effects of an integrated IFM exercise program with a 3-D printing foot core training device on SPPB, TUG test, foot pressure distribution analysis, and body composition. The Wilcoxon signed rank test was used to compare the difference before and after the exercise program. The 95% confidence ellipse area of COP under the eyes-closed condition was significantly decreased (median pretest: 217.3, IQR: 238.4; median posttest: 131.7, IQR: 199.5; *P* = 0.001) after the program (Figs. 2 and 3). All participants showed no significant differences in the SPPB and TUG tests. Regarding body composition, female fat mass significantly decreased (median pretest: 37.8, IQR: 10.4; median posttest: 37.2, IQR: 11.5; *P* < 0.001).

**Table 3:** Pre-to-post measures of integrated intrinsic foot muscle exercise program among all participants

Items (*n* = 40)	Pretest	Posttest	P-value
SPPB	12.0 (10.3, 12.0)	12.0 (11.0, 12.0)	0.15
TUG test, sec	9.0 (7.9, 11.3)	9.6 (7.9, 11.2)	0.29
95% confidence ellipse area, mm^2^ (sway area) (*n* = 39)	217.3 (144.9, 383.3)	131.7 (92.9, 292.4)	0.001*
Total sway length of COP, mm (*n* = 39)	330.0 (251.3, 486.5)	293.2 (195.4, 498.6)	0.34
Average velocity of COP, mm/sec (*n* = 39)	11.6 (8.8, 17.0)	10.3 (6.8, 17.5)	0.35
**ASMI, kg/m** ^**2**^			
Female (*n* = 32)	6.8 (6.3, 7.1)	6.8 (6.5, 7.5)	0.07
Male (*n* = 8)	9.6 (8.7, 10.3)	9.7 (9.3, 10.5)	0.33
**Fat mass, %**			
Female (*n* = 32)	37.8 (32.4, 42.8)	37.2 (30.7, 42.2)	<0.001*
Male (*n* = 8)	25.9 (22.3, 27.9)	24.9 (21.7, 27.3)	0.16

*Note* Data are presented as median (Q1, Q3)

*P < 0.05

*SPPB*, short physical performance battery; *TUG*, timed up and go; *COP*, center of pressure; *ASMI*, appendicular skeletal muscle mass index

Among participants without balance impairment, the 95% confidence ellipse area, total sway length, and average velocity of the center of pressure (COP) under the eyes-closed condition showed a significant decrease. Female participants demonstrated a significant increase in ASMI (median pretest: 6.5, IQR: 1.0; median posttest: 6.8, IQR: 1.1; *P* = 0.03) and a decrease in fat mass (median pretest: 37.8, IQR: 11.9; median posttest: 36.4, IQR: 12.5; *P* < 0.001) following the exercise program (Table 4). Among balance-impaired participants, the exercise program demonstrated significantly improved SPPB (Table 5).

**Table 4:** Pre-to-post measures of integrated intrinsic foot muscle exercise program among participants without balance impairment

Items (*n* = 29)	Pretest	Posttest	P-value
SPPB	12.0 (12.0, 12.0)	12.0 (12.0, 12.0)	0.76
TUG test, sec	8.5 (7.7, 10.6)	8.8 (7.8, 10.4)	0.17
95% confidence ellipse area, mm^2^ (sway area) (*n* = 28)	219.0 (136.7, 372.5)	122.4 (70.5, 176.4)	0.001*
Total sway length of COP, mm (*n* = 28)	335.5 (241.8, 482.2)	265.5 (178.5, 483.7)	0.02*
Average velocity of COP, mm/sec (*n* = 28)	11.7 (8.5, 16.9)	9.3 (6.3, 17.0)	0.02*
**ASMI, kg/m** ^**2**^			
Female (*n* = 22)	6.5 (6.2, 7.2)	6.8 (6.4, 7.5)	0.03*
Male (*n* = 7)	10.1 (8.6, 10.4)	9.7 (9.3, 10.6)	0.61
**Fat mass, %**			
Female (*n* = 22)	37.8 (30.6, 42.5)	36.4 (28.9, 41.4)	<0.001*
Male (*n* = 7)	25.3 (22.2, 28.1)	24.4 (20.8, 27.4)	0.24

*Note* Data are presented as median (Q1, Q3)

*P < 0.05

*SPPB*, short physical performance battery; *TUG*, timed up and go; *COP*, center of pressure; *ASMI*, appendicular skeletal muscle mass index

**Table 5:** Pre-to-post measures of integrated intrinsic foot muscle exercise program among participants with balance impairment

Items (*n* = 11)	Pretest	Posttest	P-value
SPPB	10.0 (7.0, 10.0)	10.0 (8.0, 12.0)	0.03*
TUG test, sec	11.4 (11.2, 14.3)	11.5 (10.9, 15.6)	0.93
95% confidence ellipse area, mm^2^ (sway area)	207.5 (166.5, 406.0)	246.6 (126.8, 356.1)	0.25
Total sway length of COP, mm	284.6 (270.8, 578.8)	392.1 (282.2, 871.2)	0.11
Average velocity of COP, mm/sec	10.0 (9.5, 20.3)	13.7 (9.9, 30.5)	0.11
**ASMI, kg/m** ^**2**^			
Female (*n* = 10)	7.0 (6.7, 7.2)	6.8 (6.5, 7.5)	0.86
**Fat mass, %**			
Female (*n* = 10)	38.3 (33.2, 45.4)	38.0 (33.6, 44.8)	0.24

*Note* Data are presented as median (Q1, Q3)

*P < 0.05

*SPPB*, short physical performance battery; *TUG*, timed up and go; *COP*, center of pressure; *ASMI*, appendicular skeletal muscle mass index

## Discussion

This study demonstrated that an integrated IFM exercise program with a 3-D printing foot core training device effectively improved balance and functional mobility in community-dwelling adults aged 60 and above. Participants who performed the integrated IFM exercise program showed improvements in static postural control, as measured by 95% confidence ellipse area under the eyes-closed condition. Furthermore, participants without balance impairment also exhibited improvements in total sway length and average velocity of COP. Female participants without balance impairment demonstrated a significant increase in ASMI and a decrease in fat mass after the exercise program. Among balance-impaired participants, the exercise program significantly improved SPPB.

Our study showed that exercise programs aimed at strengthening the foot’s intrinsic muscles in adults aged 60 and above might significantly improve static postural control. This finding agrees with a previous study by Spink et al., which found that older adults had improved their static balance (postural sway) following multifaceted podiatry intervention [8]. The findings of this study have two important clinical implications. First, with proper instruction and repeated practice, individuals can effectively engage in IFM training. IFM exercises pose a challenge because these muscles usually require dedicated training. In our study, we introduced an integrated exercise program with a novel foot core training device, accompanied by instructional videos. These modifications aimed to reduce the difficulty and enhance the feasibility of the exercise program for all study participants. Additionally, our findings suggest that older adults can safely perform the IFM exercise program (most of the program is seated) as an independent exercise program, leading to changes in balance in as little as 4 weeks. However, for individuals new to the training, we recommend having a medical or health specialist available to provide initial instruction on correctly performing the IFM exercise program. This ensures a safe and effective program commencement for participants who may need to become more familiar with the exercises.

Our study applied foot pressure distribution analysis instead of single IFM morphology to measure the effect of the exercise program. A previous meta-analysis revealed no significant difference in IFM morphology parameters post-training, encompassing IFM thickness, cross-sectional area, and volume [23]. The constrained volume of IFMs presents challenges in detecting subtle changes in foot muscles [24]. Furthermore, considering the diverse origins and insertions of IFMs, interventions may result in different levels of engagement, adding complexity to the identification of uniform changes [25, 26]. Therefore, to overcome these challenges, we have opted for alternative measures. Foot pressure distribution analysis emerges as a more suitable method for gauging the impact of interventions on postural control in older adults [9, 27]. Sway area and total sway length are two common postural control outcome variables calculated from force platform data, which differ from generic balance assessment (e.g., Berg Balance Scale) and allow for a better understanding of mechanisms that may improve balance performance [27].

A proposed mechanism for improved balance control due to IFM training is that the sensorimotor function of plantar IFMs following the exercise program may be improved; this allows the body to limit postural sway to a smaller area and reduce movement length and velocity, thereby decreasing the need for postural corrections of large movements [12, 27]. Balance-impaired participants in our study significantly improved SPPB measures, mainly in static balance scores, further supporting this theory. As important local foot stabilizers and sensors, IFMs work in concert with the extrinsic muscles of the ankle-foot complex in the foot core system [4]. We integrated the isolated intrinsic foot strengthening exercises with ankle strengthening exercises, which may also result in extrinsic and intrinsic motor changes. Although it is difficult to determine the extent to which each exercise, alone or in combination, affects the outcome of foot control, an integrated exercise program emphasizing the IFMs exercise may be more effective due to the complex interactions in the foot core system.

The observed improvement in ASMI and reduction in fat mass following the exercise program for participants without balance impairment can be attributed to the engagement of lower limb and leg muscles, including the quadriceps and gastrocnemius, during integrated exercises. While performing active ankle exercises, participants, seated in a chair, were instructed to utilize their quadriceps to elevate their feet and facilitate ankle movement. Bilateral standing calf raises predominantly activate the gastrocnemius muscle at the back of the calf.

A strength of this study was its quasi-experimental single-group pretest-posttest design, which helped trace nearly all community-dwelling adults aged 60 and above in the community care center. A total of 76.9% of eligible participants successfully completed the exercise program, demonstrating a median adherence rate of 88.9%. In addition, integrated foot core strengthening exercises with a 3-D printing foot core training device for balance, functional mobility, and sarcopenia prevention in older adults were feasible in the community. Moreover, we employed various well-established functional outcomes, including SPPB and foot pressure distribution analysis, enabling a comprehensive evaluation of the exercise program’s impact on both balance and mobility.

Notably, this study has some limitations. First, this was a preliminary study with a relatively small sample size, and the exercise program was limited to a 4-week program. However, we used objective measurements such as foot pressure distribution to identify the training effect of IFMs in our research, suggesting that the effect of an integrated IFM exercise program was robust and promising for geriatric rehabilitation. Due to the high variability of baseline functional mobility and balance, future studies with larger cohorts and extended program periods are warranted to demonstrate a more significant training effect. Additionally, IFM exercises were only supervised in the community care center; thus, we could not confirm continued practice after participants returned home. Nonetheless, we used daily training logs to assess compliance and recorded a complete instructional video for participants to reference. This strategy likely increased adherence and decreased the difficulty perceived by older adults. Lastly, this study did not record the direct outcome of falls, which was an important geriatric syndrome. Still, the findings that the integrated IFM exercise program improved balance may decrease the risk of falls in older adults [9]. This assumption needs further research to verify.

## Conclusion

The 4-week integrated IFM exercise program, coupled with a 3-D printing foot core training device, demonstrates notable enhancements in static balance among older adults. Importantly, there were significant improvements in body composition observed among participants without balance impairment and improved functional mobility seen in those with balance impairment. These findings underscore the efficacy of integrated IFM strengthening exercises as beneficial programs for enhancing balance and body composition in older adults.

## Data Availability

The datasets generated and analyzed during the study may be made available by the corresponding author upon reasonable request.

## Abbreviations

ADLs: Activities of daily living
ASM: Appendicular skeletal muscle mass
ASMI: ASM index
COP: Center of pressure
IFMs: Intrinsic foot muscles
IQR: Interquartile range
SFEs: Short foot exercises
SPPB: Short physical performance battery
TUG: Timed up and go
